# Predicting Therapeutic Response to Antibody-Drug Conjugates Using Targeted PET Imaging: A Systematic Review

**DOI:** 10.3390/cancers18152514

**Published:** 2026-08-05

**Authors:** David Mourath, Nour Susaeg Romdhani, Viveka Bergman, Jonathan Siikanen, Thuy A. Tran, Ali Alhuseinalkhudhur, Anna Kistner, Renske Altena

**Affiliations:** 1 Department of Oncology, Linköping University Hospital, 581 85 Linköping, Sweden; david.mourath@regionostergotland.se (D.M.);; 2 Department of Oncology-Pathology, Karolinska Institutet, 171 77 Stockholm, Sweden; 3 Department of Nuclear Medicine and Medical Physics, Karolinska University Hospital, 171 76 Stockholm, Sweden; 4 Theranostics Trial Center, Department of Nuclear Medicine and Medical Physics, Karolinska University Hospital, 171 76 Stockholm, Sweden; 5 Nuclear Medicine and PET, Department of Surgical Sciences, Uppsala University, 753 10 Uppsala, Sweden; 6 Department of Molecular Medicine and Surgery, Karolinska Institutet, 171 77 Stockholm, Sweden

**Keywords:** antibody-drug conjugate, targeted PET, immuno-PET, response prediction, predictive biomarker, theranostics, HER2, Nectin-4

## Abstract

Background: Antibody-drug conjugates (ADCs) are gaining rapidly expanding clinical utility, with a growing number of approved indications across compounds that differ in target antigen, payload mechanism, and linker design. However, treatment response remains heterogeneous, and predictive biomarkers to identify patients most likely to benefit are limited. Given that ADC efficacy depends on sufficient target antigen expression and distribution across tumor lesions, positron emission tomography (PET) imaging offers a unique opportunity to non-invasively assess whole-body target availability. We therefore conducted a systematic literature review to evaluate the relationship between PET-measured tumor antigen expression and the therapeutic response to ADCs targeting the same antigen. Method: A systematic comprehensive search of PubMed, EMBASE and Web of Science databases was conducted in accordance with Preferred Reporting Items for Systematic Reviews and Meta-Analyses (PRISMA) guidelines, using terms related to targeted PET, ADC treatment and response assessment. Article screening, eligibility assessment, and data extraction were performed independently using predefined criteria by two reviewers. Disagreements were resolved by consensus. Results: A total of 5628 records were identified. After removing duplicates, 4323 records were screened by title and abstract. Fifty-eight underwent full-text review and seven were included in the final review. Four trials investigated human epidermal growth factor receptor 2 (HER2)-targeted therapy, one Nectin-4, one mesothelin and one STEAP1. In all HER2- and Nectin-4-related trials, patients with a higher uptake on targeted PET had a higher probability of responding to targeted treatment. Included trials were generally small and had a moderate risk of bias. Conclusion: Targeted PET imaging showed potential to predict treatment response in trials evaluating clinically active agents. Additional clinical trials across a broader range of targets, along with standardized acquisition protocols and harmonized study designs, are needed to enable the integration of this technique into clinical practice and ultimately translate its benefits to patients.

## Simple Summary

Antibody-drug conjugates (ADCs) are a rapidly growing class of cancer drugs that deliver a potent toxic payload directly to tumor cells carrying a specific target antigen. However, not all patients respond, and there is currently no reliable way to identify in advance who will benefit. Positron emission tomography (PET) using tracers directed at the same antigen can non-invasively show how much of the target is present throughout the whole body. In this systematic review, we examined whether such targeted PET imaging can predict response to ADC treatment. Across seven small clinical trials, higher PET uptake was generally associated with a greater likelihood of responding, particularly for HER2− and Nectin-4-targeted therapies. These findings suggest targeted PET could help select patients for ADC therapy, but larger, standardized studies are needed before clinical use.

## 1. Introduction

**Figure 1:**
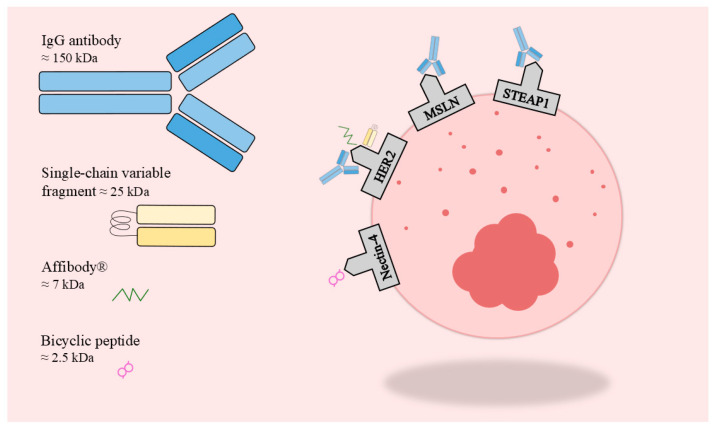
Ligands and targets used for PET and ADC-treatment in included trials. HER2 = human epidermal growth factor receptor 2, MSLN = membrane-bound surface glycoprotein mesothelin, STEAP1 = Six-transmembrane epithelial antigen of the prostate 1.

The increasing use of targeted therapies has transformed cancer treatment by improving treatment outcomes while reducing off-target toxicity compared with traditional chemotherapy-based regimens [1,2]. The unraveling of cellular signal transduction pathways has paved the way for the development of highly specific therapeutic agents aimed at addressing the hallmarks of cancer [3,4]. Currently, the primary therapies utilizing this approach are monoclonal antibodies and tyrosine kinase inhibitors [5]. Antibody-drug conjugates (ADCs) have emerged as a rapidly expanding class of agents that offer targeted treatments that combine the high specificity of vectors such as monoclonal antibodies, with the potent cytotoxic activity of highly effective payloads. ADCs rely on selective binding to cell-surface antigens, followed by internalization and intracellular payload release, thereby enabling targeted delivery to the malignant cells with reduced damage to healthy tissues [6]. As of May 2026, there are 21 approved ADCs on the global market targeting 13 different antigens (Figure 1, Table 1) and over 400 ADCs are in Phase I, II, and III clinical trials involving 53 different targets. Of these 53 targets, five (human epidermal growth factor receptor 2 (HER2), CD30, B-cell maturation antigen (BCMA), Nectin-4, and CD19) account for the majority of active trials [7]. Although targeting the same antigen, different ADCs can utilize a myriad of different payloads as well as varying linkers and even targeting ligands. This makes the efficacy, safety and pharmacokinetics of each agent distinct.

Most oncological treatments still lack sensitive and specific biomarkers that predict treatment efficacy, and if present, often only tissue-based samples from a single tumor biopsy are used. Targeted PET, herein defined as PET imaging with radiolabeled vectors designed to bind tumor-associated molecular targets, enables whole-body in vivo imaging of specific tumor biomarkers, such as those targeted by ADCs and radioligand therapies [8]. When antibody-derived ligands are used, this approach is commonly referred to as immuno-PET [9]. In radioligand therapy (RLT), a targeting vector such as a peptide, small molecule, antibody or antibody fragment, is coupled to a therapeutic radionuclide to deliver cytotoxic radiation to cells expressing a specific molecular target, and the same or a closely related vector can instead be labeled with a positron-emitting radionuclide for PET imaging. The theranostic principle “Treat what you see, see what you treat” is well established in RLT [10]. Treatment with [^177^Lu]Lu-Dotatate (LUTATHERA^®^) as well as [^177^Lu]Lu-PSMA-617 (PLUVICTO^®^) is preceded by receptor-specific PET to confirm target expression [11,12]. In neuroendocrine tumors, somatostatin-receptor imaging with [^68^Ga]Ga-DOTATATE PET selects patients for [^177^Lu]Lu-DOTATATE, and in prostate cancer PSMA-ligand PET precedes [^177^Lu]Lu-PSMA-617; in both settings the diagnostic and therapeutic agents share the same targeting vector, so that tracer uptake directly reflects the molecular target engaged by the therapy. This differs from several ADC imaging studies that use surrogate vectors, a distinction relevant to the interpretation of the present review (see Limitations). Several trials have revealed a correlation between target expression and treatment outcome in the case of RLT [13,14]. ADCs share many similarities with RLTs: in both systems, a targeting vector aims to recognize tumor-associated antigens and deliver a linked anti-neoplastic payload [15]. There is a biological rationale for the assumption that high target expression correlates with increased drug delivery to tumor cells and with therapeutic response [16,17]. ADC therapy is associated with clinical and financial toxicity, and accurately predicting treatment response would benefit patients by guiding appropriate therapy and avoiding ineffective treatment. Conventional biopsies of individual lesions capture only a small fraction of an often heterogeneous disease whereas whole-body targeted PET provides a more comprehensive representation of antigen expression in metastatic cancers [18].

The aim of this systematic review is to investigate whether tumor antigen expression visualized by targeted PET correlates with the therapeutic efficacy of ADCs targeting the same antigen. For this purpose, we reviewed the available literature on the capability of target-specific PET imaging to predict response to ADC treatment.

## 2. Material and Methods

We conducted a comprehensive search in the PubMed, EMBASE and Web of Science databases up to 8 June 2026 following the Preferred Reporting Items for Systematic Reviews and Meta-Analyses (PRISMA) guidelines (Table S1). Search terms varied slightly between databases. Search strategies for the EMBASE and Web of Science databases are provided in Supplemental Table S2; the search strategy used for PubMed is presented here:

(“Neoplasms”[Mesh] OR cancer) **AND** (“Positron-Emission Tomography”[Mesh] OR “Positron Emission Tomography Computed Tomography”[Mesh] OR PET OR PET-CT) **AND** (“Immunoconjugates”[Mesh] OR “Immunoconjugates” [Pharmacological Action] OR “Antibody drug conjugates” OR ADC OR T-DM1 OR T-DXd OR Gemtuzumab ozogamicin OR Mylotarg OR Brentuximab vedotin OR Adcetris OR Trastuzumab emtansine OR Kadcyla OR Inotuzumab ozogamicin OR Besponsa OR Moxetumomab pasudotox OR Lumoxiti OR Polatuzumab vedotin OR Polivy OR Enfortumab vedotin OR Padcev OR Trastuzumab deruxtecan OR Enhertu OR Sacituzumab govitecan OR Trodelvy OR Belantamab mafodotin OR Blenrep OR Loncastuximab tesirine OR Zynlonta OR Tisotumab vedotin OR Tivdak OR Mirvetuximab soravtansine OR ELAHERE OR Disitamab vedotin OR Aidixi OR Cetuximab sarotalocan OR Akalux) Filters: **English, Humans**.

Article screening, eligibility assessment, extraction and interpretation of data were done and verified by two reviewers (DM, NSR). Any disagreements were resolved through discussion until consensus was reached. Included articles were read in full, and publication journal, author, year, sample size, treatment characteristics, diagnostic modalities and disease and patient outcomes, were extracted. All data was organized in Microsoft Excel. The PROSPERO registration number for this review is CRD42024624089. The data were compiled, synthesized descriptively, and interpreted through narrative synthesis.

The PICO framework was applied to screen the results: Population (Patients with cancer receiving ADC therapy), Intervention (Targeted PET performed, utilizing a tracer targeting the same antigen as the ADC), and Outcome (Accuracy of targeted PET in predicting therapeutic response to ADCs). No relevant comparison element was identified. The complete PICO statement can be viewed in Supplemental Table S3 [19].

Risk of bias assessment was done using the QUIPS (Quality In Prognosis Studies) tool [20].

## 3. Results

**Figure 2:**
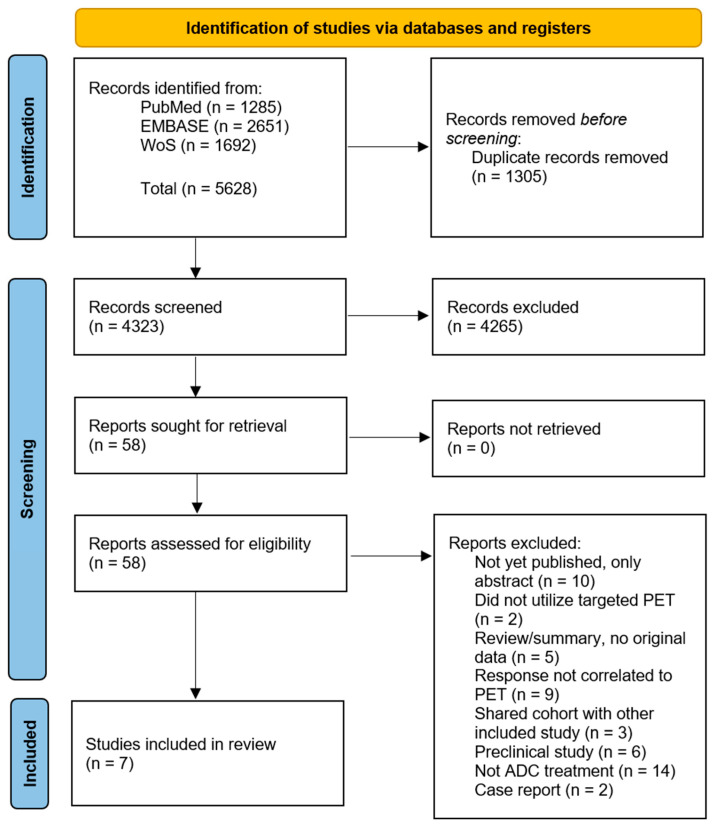
Flow-diagram describing screening and inclusion in systematic review. ADC = Antibody-drug conjugate.

A total of 5628 records were retrieved through the literature search. After the removal of duplicates, 4323 records remained and were screened by title and abstract. Of these, 58 articles were selected for full-text assessment. Seven trials met the inclusion criteria and were included in the review (Figure 2).

Data from 188 patients included in seven clinical trials contributed to the dataset. Four trials with a total of 152 patients examined HER2-targeted ADCs, one trial with 10 patients evaluated a Nectin-4-targeted ADC, one with 11 patients evaluated an MSLN-targeted ADC, and one with 15 patients assessed a STEAP1-targeted ADC.

### 3.1. Trials Concerning HER2-Targeted ADCs

Trastuzumab emtansine (T-DM1) was the most frequently investigated drug, evaluated in three of the four trials on HER2-targeted therapies. Reporting the final results of the ZEPHIR trial, Mileva et al. [21] used [^89^Zr]Zr-trastuzumab-PET to identify lesions and patients unlikely to respond to treatment in a cohort of 83 patients, the largest cohort identified in this review. The reported negative predictive value was 84% (95% CI 64–95%). Median time to treatment failure (TTF) differed significantly between PET-positive and PET-negative patients, with median TTFs of 9.9 months and 2.8 months, respectively. In a smaller pilot study, Mortimer et al. [22] performed [^64^Cu]Cu-trastuzumab-PET in 10 patients treated with T-DM1. Patients with high PET uptake experienced a markedly longer TTF: 28 versus 2 months. In 2023, Alhuseinalkhudhur et al. [23] published an interim analysis of a larger phase 2 and 3 prognostic trial utilizing the ^68^Ga-labeled affibody ABY-025 to perform targeted PET in 21 patients with metastatic breast cancer. PET imaging was able to predict a lack of response to various HER2-directed therapies, including T-DM1.

Lee et al. [24] explored the ability of PET uptake to predict response to MM-302 (a HER2-targeted pegylated antibody-liposomal doxorubicin conjugate) that was itself radiolabeled with ^64^Cu, in 19 patients with metastatic breast cancer. No formal statistical analysis was performed, but a numerical trend was observed in which 75% of patients in the high deposition group, where more of the drug reached the tumor, responded to therapy compared to 43% in the low deposition group.

**Figure 3:**
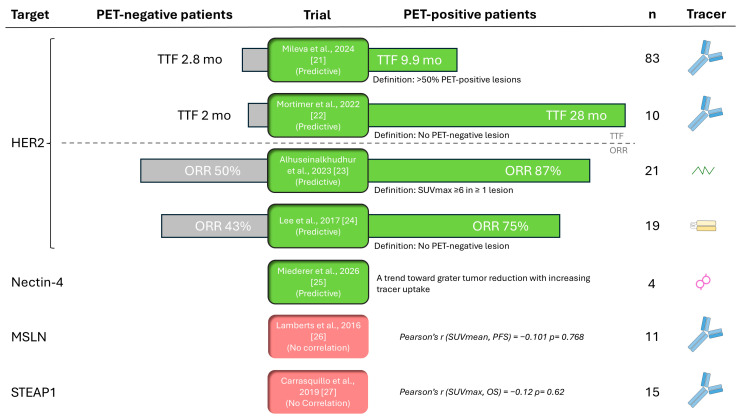
Relation between targeted PET uptake and treatment response to ADC targeting the same antigen. TTF = Time to Treatment Failure, ORR = Overall Response Rate, STEAP1 = Six-transmembrane epithelial antigen of the prostate 1, MSLN = membrane-bound surface glycoprotein mesothelin, PFS = Progression free survival, OS = Overall survival [21,22,23,24,25,26,27].

Across these trials, PET uptake consistently emerged as a predictive indicator of treatment response, as shown in Table 2 and Figure 3.

**Table 2:** Trials evaluating therapeutic response to antibody-drug conjugates in relation to uptake on targeted PET.

Author, Year (Ref)	Summary Therapy Prediction Results	Phase	Patient				Treatment			PET			Evaluation			
			*n*	Cancer Type	ECOG	Prior Therapy Lines	Name	Target	Dose and Treatment Intervals	Targeting Vector	Radionuclide	MBq Injected	Outcome Measure (Evaluation Modality)	Time to Evaluation	Definition of PET-Positive Lesion	Definition of PET-Positive Patient
Mileva et al., 2024 [21]	Patient: PPV 77% (95% CI 64–88%), NPV 84% (95% CI 64–95%) Lesion: PPV 72% (95% CI 64–78%), NPV 81% (95% CI 71–88%) TTF 9.9 vs. 2.8 months	II	83	Breast	0–1	0–11+	T-DM1	HER2	3.6 mg/kg in 3-week intervals	Trastuzumab	^89^Zr	37 ± 10%	Anatomical response + TTF (CT, RECIST 1.1)	3 treatment cycles (9 weeks)	Qualitative comparison to healthy tissue	Dominant part of lesions determined as PET-positive
Mortimer et al., 2022 [22]	TTF 28 vs. 2 months, HR 0.1 (95% CI 0.1–1.0)	0	10	Breast	0–2	NR	T-DM1	HER2	3.6 (8 patients) or 2.7–3.0 (2 patients) mg/kg 3-week intervals.	Trastuzumab	^64^Cu	475–606	TTF (FDG-PET, PERCIST)	2 treatment cycles (6 weeks)	SUVmax ≥ 5.5 g/mL	No PET-negative lesion
Alhuseinalkhudhur et al., 2023 [23]	PBC + MBC: Sensitivity 56%, Specificity 66% MBC: Sensitivity 71%, Specificity 67% Soft-tissue lesions: Sensitivity 86%, Specificity 67% Skeletal lesions: Sensitivity 69%, Specificity 83%	II	40 **	Breast	0–2	0–6+	TZB, Pertuzumab, Chemotherapy and T-DM1	HER2	NR	HER2-targeted Affibody	^68^Ga	139 ± 43	Metabolic response, delta-TLG lower than −25%. (FDG-PET, measured in the 5 largest lesions)	2 treatment cycles (6 weeks)	Soft tissue: SUVmax ≥ 6 Skeletal lesions: SUVmax ≥ 16.2	SUVmax ≥ 10.7 (all patients) or ≥10.9 (MBC) in at least one lesion
Lee et al., 2017 [24]	PR/SD: 75% vs. 43% PFS 2.0 vs. 1.7 months	I *	19	Breast	0–1	NR	MM-302 + TZB with or without CTX	HER2	MM-302: 30 mg/m^2^. TZB: 6 mg/kg, some interpatient variation. CTX: 450 mg/m^2^ (10 patients) Every 3 weeks.	Single-chain variable fragment (scFv)	^64^Cu	337–432	Anatomical response + PFS (CT, RECIST 1.1)	Every 8 weeks	%-injected dose/kg ≥ 2	No PET-negative lesion
Lamberts et al., 2016 [26]	Mean SUVmax did not correlate to PFS or anatomical response.	I *	11	Pancreas (*n* = 7) Ovarian (*n* = 4)	0–1	NR	DMOT4039A	MSLN	0.8–1.2 mg/kg every week (5 patients). 2.4–2.8 mg/kg in 3-week intervals (6 patients).	MSLN-targeted IgG1 monoclonal antibody	^89^Zr	36.78 ± 1.26	Anatomical response + PFS (CT, RECIST 1.1)	2 treatment cycles (2 and 6 weeks)	NA	NA
Carrasquillo et al., 2019 [27]	SUVmax did not correlate to OS, nadir PSA, or time on study drug.	I/II	15	Prostate	KPS ≥ 60%	1–8	DSTP3086S	STEAP1	Dose escalation study: 0.3 to 2.8 mg/kg in 3-week intervals	STEAP1-targeted IgG1 monoclonal antibody	^89^Zr	170–199	PSA, OS, Time on study drug (Blood samples)	NR	NA	NA
Miedere et al., 2026 [25]	Trend towards greater change in tumor volume with increasing SUVmean.	I	4	Urothelial cancer	NR	0–2	EV/P	Nectin-4	NR	Bicyclic peptide	^68^Ga	1.6 ± 0.5/kg	% change in tumor volume (CT)	12 weeks	NA	NA

CT = Computer tomography, CTX = Cyclophosphamide, DMOT4039A = Anti-MSLN IgG1 antibody and monomethyl auristatin (MMAE), DSTP3086S = Ant-STEAP1 IgG1 antibody and monomethyl auristatin (MMAE), ECOG = Eastern Cooperative Oncology Group, EV/P = Enfortumab vedotin plus pembrolizumab, KPS = Karnofsky Performance Score, MBC = Metastatic breast cancer, MM-302 = HER2-targeted PEGylated antibody-liposomal doxorubicin conjugate, MSLN = membrane-bound surface glycoprotein mesothelin, NA = Not applicable, NPV = Negative predictive value, NR = Not Reported, OS = Overall survival, PET = Positron emission tomography, PFS = Progression free survival, PPV = Positive predictive value, PR = Partial response, PSA = Prostate-specific antigen, SD = Stable disease, STEAP1 = Six-transmembrane epithelial antigen of the prostate 1, T-DM1 = Trastuzumab emtansine, TLG = SUVmean × metabolic tumor volume, TTF = Time to treatment failure, TZB = Trastuzumab, * = Substudy, ** = A total of 39 patients received anti HER2-therapy however, the specific number of patients treated with T-DM1 are not disclosed. PBC= Primary breast cancer.

### 3.2. Trials Concerning Nectin-4 Targeted ADC

One trial on targeted PET in urothelial cancer treated with enfortumab vedotin plus pembrolizumab (EV/P) was identified. Miederer et al. [25] investigated whether Nectin-4-targeted PET using a ^68^Ga-labeled bicyclic peptide was associated with treatment response. Although 10 patients were included, only four could be evaluated for response to therapy. Altogether, eight metastatic lesions were evaluated, and the authors noted a trend toward a greater change in tumor volume with increasing SUVmean. No statistical analysis was carried out. Therapy with EV/P was highly active in this small cohort, with all measured metastases decreasing in volume by more than 70%. No data on TTF or survival is presented.

### 3.3. Trials of Emerging Targets for ADC’s

Two trials investigated whether targeted PET could predict treatment response with MSLN- and STEAP1-targeted therapies, respectively. In 2016, Lamberts et al. [26] published a study in conjunction with a phase 1 trial evaluating an anti-MSLN IgG1 antibody and monomethyl auristatin conjugate, enrolling 11 patients with pancreatic cancer (*n* = 7) or ovarian cancer (*n* = 4). Tumor biopsies and IHC for MSLN were acquired, but a positive stain was not required for inclusion. In 2019, Carrasquillo et al. [27] published an imaging study conducted alongside a dose-escalation trial of a STEAP1-targeted ADC in metastatic prostate cancer. In this cohort, 15 patients underwent targeted PET before treatment started and were evaluated for response in relation to tracer uptake.

Neither trial demonstrated an ability to predict treatment response. However, both trials were limited by small sample sizes and evaluated novel ADCs with uncertain efficacy.

### 3.4. Risk of Bias Assessment

The QUIPS (Quality In Prognosis Studies) tool identifies six important domains when evaluating bias: study participation, study attrition, prognostic factor measurement, outcome measurement, study confounding, and statistical analysis and reporting [20]. It should be noted that the reported risk of bias in Table 3 is in relation to the specific research question of predicting therapeutic response, or lack thereof, to ADC treatment using targeted PET. This was not necessarily the primary aim of the included trials, as in the case of the trial by Alhuseinalkhudhur et al. in which response to all kinds of HER2-directed therapy was evaluated.

Several trials highlighted that low participant numbers limit the strength of any conclusions, as reflected in the QUIPS domain “study participation”. The substantial heterogeneity in design and treatments across the included trials made meta-analysis unlikely to be informative. Adjustments for confounders, such as age, performance status, and prior treatment lines, were limited across trials. This may be relevant, as Alhuseinalkhudhur et al. identified prior lines of therapy as a marker of outcome [23].

Most papers clearly reported drug dose, administered activity, and administration protocol (Table 2). However, several trials introduced variability in treatment exposure that complicated the interpretation of treatment response. This is captured in the domain “prognostic factor measurement” in the QUIPS tool. Three of the HER2 trials [22,23,24] and the MSLN trial [26] included multiple treatment regimens and/or dose levels without presenting response outcomes stratified by regimen or dose. In the STEAP1-trial [27], patients were treated at different dose levels, and 6 out of 15 patients discontinued treatment before evaluation due to adverse effects, which was not reported as accounted for in the response assessment.

## 4. Discussion

There is an unmet clinical need for improved therapy-predictive biomarkers to identify patients likely to benefit from treatment with an ADC. A targeted PET-based approach may, based on common practices with RLT given the structural similarities between RLT and ADCs, provide a promising therapy-predictive tool. In this systematic literature review, we identified and synthesized seven clinical trials evaluating the predictive capability of targeted PET, including immuno-PET, in relation to ADC therapy. A targeted PET-based treatment selection approach offers the potential to spare patients unlikely to benefit from therapy from unnecessary toxicity and enables treatment to be offered to patients who might otherwise not have been considered eligible. For currently used ADCs such as trastuzumab emtansine and enfortumab vedotin, a clear trend was observed where patients exhibiting higher radiotracer uptake demonstrated better treatment outcomes. Several trials included in this review found no clear correlation between IHC status and treatment response. This may be explained by tumor heterogeneity, staining protocols, and temporal changes in expression. Alhuseinalkhudhur et al. reported four patients with metastatic breast cancer who achieved complete responses to HER2-targeted therapy despite being HER2-negative on biopsy [23]. This finding underscores the predictive value of targeted PET and highlights the inherent limitations of localized, tissue-based assessments.

### Defining PET-Positivity

One aspect that would benefit from unified definitions is what should be considered a “PET-positive” lesion and, by extension, patient [22]. There are two fundamentally different approaches described in the literature. Either a lesion is defined as positive in relation to its IHC grade, or positivity is defined as the point at which a therapeutic agent has a measurable effect [28,29]. In the latter case, different cutoffs must be considered for different drugs depending on their potency and/or tracer ligand affinity and binding. An analogy can be drawn to the fact that T-DM1 and T-DXd require different HER2 IHC cutoffs to be regarded as effective treatment options [1,30]. Most included trials proposed SUV cutoff values to predict therapeutic response in this manner. However, these cutoffs are not directly comparable, as trials employed different isotopes and treatment protocols. Mortimer et al. [22] identified an SUVmax threshold of ≥5.5 as the optimal cutoff for predicting patient response. Notably, one proposed cutoff by Alhuseinalkhudhur et al. [23] was very similar, at SUVmax ≥ 6. Mileva et al. [21] used a qualitative approach in which SUV was compared with healthy tissue within the same organ as the lesion of interest, whereas Miederer et al. [25], Lamberts et al. [26], and Carrasquillo et al. [27] treated SUVmax/mean as continuous variables. Lee et al. [24] measured the percent of injected tracer dose per kg of tissue instead of SUV. An additional consideration is that the optimal SUV cutoff for predicting treatment response may vary according to the tissue site of the metastasis. One study found that the optimal SUVmax cutoff to predict metabolic response in skeletal lesions was significantly higher (16.2) than the value proposed for soft tissue lesions (6.0). Both cutoffs yielded comparable discrimination (AUC 0.74 versus 0.81) for the respective tissue sites [23].

Of key clinical relevance is determining whether a patient is regarded as PET-positive and therefore a potential candidate for treatment. Several different approaches are represented within the included studies. In one analysis by Alhuseinalkhudhur et al. [23] patient-level positivity was determined by the SUVmax of one biopsied lesion. In contrast, Mileva et al. and the ZEPHIR trial [21] required that the majority of lesions be positive for a patient to qualify as PET positive. Mortimer et al. [22] and Lee et al. [24] applied even stricter criteria, requiring all lesions to be PET positive for the patient to be classified as positive. For most of these considerations, it is essential to have a clear understanding of what constitutes a true cancer lesion. Otherwise, there is a risk of misclassifying a benign lesion as a PET-negative metastasis and thereby withholding effective treatment or, conversely, overinterpreting false-positive uptake on targeted PET and prescribing ineffective therapy to patients who should be offered alternative options.

When evaluating these cutoffs, the intrinsic limitations of PET imaging must be considered. Test–retest studies on [^18^F]FDG-PET have reported inter-test differences of 10% [31]. Lesion size relative to the spatial resolution of the PET system may substantially influence measured uptake due to partial volume effects [32]. Consequently, tracer uptake can be underestimated in small lesions. SUVmax was chosen as the quantitative measure in the majority of the included trials and is a simple and clinically useful metric, but it may, as it is based on a single voxel, be particularly susceptible to image noise and statistical fluctuations [33]. A viable alternative could be SUV peak, defined as the average uptake within a small, fixed-size region, combining the reproducibility of SUVmax with the robustness of SUVmean, without the need for individual lesion segmentation. The choice of metric should be guided by imaging characteristics, lesion size, and intended application. Quantification of receptor expression using PET may also be influenced by inter-examination variability in tracer pharmacokinetics, biodistribution, and patient-specific physiological factors. Furthermore, physiological tracer uptake in the liver as well as in other reference organs such as the spleen and blood pool, can vary between PET scans, introducing potential confounders. Comparing tracer uptake in normal liver tissue within the same individual may help assess changes between baseline and post-treatment scans. Consequently, differences in tumor uptake between examinations may not solely reflect changes in receptor expression, and normalization strategies may improve comparability between scans. Normalization approaches have been explored in ^89^Zr-immuno-PET as well as in HER2 imaging with the affibody ABY-025 [34,35].

## 5. Limitations

Included trials generally had a high risk of bias in key domains, such as confounders and differences in treatment regimens between individual participants, without these differences accounted for in the response assessment. Furthermore, included trials are heterogeneous and vary in design and methodology regarding treatment regimens, choice of diagnostic vector, and isotopes linked to these vectors. An important consideration is that not all imaging vectors evaluated in this review targeted the same epitope as the corresponding ADC. Several studies used surrogate targeting molecules, such as affibodies or bicyclic peptides, rather than the therapeutic antibody itself. While these agents may offer favorable imaging characteristics, their uptake may not accurately reflect ADC binding or delivery if epitope specificity differs. This, in addition to the generally low number of participants, limits the insights gained from the available literature. The choice of targeting vector is a central determinant of what targeted PET actually measures, and vector size entails important trade-offs. Small vectors such as affibodies, single-chain variable fragments and bicyclic peptides clear rapidly from the blood, achieve high tumor-to-background contrast and allow same-day imaging with short-lived radionuclides such as [^68^Ga] or [^18^F], while delivering a lower radiation dose; their smaller size may also improve tumor penetration. However, they may recognize a different epitope, and often with different affinity and avidity, than the full-length therapeutic antibody, so their uptake may not faithfully reflect the binding, internalization and payload delivery of the corresponding ADC, and rapid renal clearance can increase background uptake in the kidneys. Intact antibody-based tracers more closely mirror the pharmacokinetics and target engagement of an ADC but require long-lived radionuclides such as [^89^Zr], entail higher radiation exposure and require delayed, multi-day imaging. The optimal vector therefore depends on whether the aim is to visualize target expression per se or to predict delivery of a specific ADC, which should be considered in the design of future studies. Targeted PET showed a predictive value in all four HER2-targeted therapy trials as well as a positive trend in the Nectin-4 trial, but not in the trials of MSLN- or STEAP1-targeted therapies. One possible explanation is that HER2 and Nectin-4-targeted therapies, such as T-DM1 and enfortumab vedotin, have established clinical efficacy, whereas the agents used in the MSLN and STEAP1 trials have not been validated and are still in development. Future trials should assess targeted PET for clinically validated ADCs targeting different antigens.

Key unresolved questions include how to define a tracer-positive lesion and, consequently, how to classify a tracer-positive patient in a clinically meaningful way. There are several strengths to the method adopted by Mileva et al., as it looks at more than a single lesion’s PET-status while minimizing the risk of withholding effective treatment due to a misinterpreted false-negative lesion by requiring only a majority of lesions to be PET-positive. IMPACT-MBC (NCT01957332) is a prospective trial enrolling more than 200 patients, set to be completed in 2027, that aims to investigate the clinical utility of targeted PET and the impact of its use on survival. The consortium conducting the trial has already published data on utilizing 16α-[18F]fluoro-17β-estradiol positron emission tomography (FES-PET) in patients with estrogen receptor-positive breast cancer metastases to predict response to endocrine therapy [36,37]. They showed that patients in whom all metastases had a SUVmax ≥ 1.5 experienced longer progression-free survival compared with patients who had heterogeneous FES-PET lesion uptake. Similarly, HER2-Ex-PET (NCT06830382) is a prospective, multicenter, phase II trial investigating the potential of [^68^Ga]Ga-ABY-025 to predict therapeutic response to T-DXd in patients with metastatic breast cancer and is currently recruiting patients across several Swedish university hospitals. These and similar trials are expected to provide results which may further help clarify how these new tools can be used in clinical practice.

## 6. Conclusions

In conclusion, PET-guided strategies to match the right patient to the right ADC at the right time are promising as therapy-selection tools, offering real-time confirmation of target presence and distribution by visualizing the same target with a diagnostic PET-based approach. Realizing their full potential will require additional clinical trials across a broader range of targets, along with standardized acquisition protocols and harmonized study designs and endpoints. With these elements in place, a “treat what you see, see what you treat” paradigm for antibody-drug conjugates may become achievable in the coming years.

## Figures and Tables

**Table 1:** Antibody-drug conjugates with EMA, NMPA and/or FDA approval.

Antibody-Drug Conjugate (Trade Name)	Target	Cytotoxic Drug	Indication	Approval Year (Medical Agency)
Gemtuzumab ozogamicin (Mylotarg^®^)	CD33	Calicheamicin	Acute myeloid leukemia	2000 (FDA) 2018 (EMA)
Brentuximab vedotin (Adcetris^®^)	CD30	MMAE (Monomethyl auristatin E)	Hodgkin’s lymphoma Systemic anaplastic large cell lymphoma Cutaneous T-cell lymphoma	2011 (FDA) 2012 (EMA)
Trastuzumab emtansine (Kadcyla^®^)	HER2	DM1	HER2+ breast cancer	2013 (FDA/EMA)
Inotuzumab ozogamicin (Besponsa^®^)	CD22	Calicheamicin	B-cell precursor acute lymphoblastic leukemia	2017 (FDA/EMA)
Moxetumomab pasudotox (Lumoxiti^®^)	CD22	PE38	Hairy cell leukemia	2018 (FDA/EMA)
Polatuzumab vedotin (Polivy^®^)	CD79	MMAE	Diffuse large B-cell lymphoma	2019 (FDA) 2020 (EMA)
Enfortumab vedotin (Padcev^®^)	Nectin-4	MMAE	Urothelial cancer	2019 (FDA) 2022 (EMA)
Trastuzumab deruxtecan (Enhertu^®^)	HER2	DXd	HER2+/low breast cancer Non-small cell lung cancer HER2+ gastric cancer Tumor agnostic approval by FDA	2019 (FDA) 2021 (EMA)
Cetuximab sarotalocan (Akalux^®^)	EGFR	700DX dye: laser activatable agent	Head and neck squamous cell carcinoma	2020 (PMDA)
Belantamab mafodotin (Blenrep^®^) *	BCMA	MMAF	Multiple myeloma	2020 (FDA/EMA)
Sacituzumab govitecan (Trodelvy^®^)	Trop-2	SN-38	HER2− breast cancer	2020 (FDA) 2021 (EMA)
Disitamab vedotin (Aidixi^®^)	HER2	MMAE	Urothelial carcinoma HER2+ gastric cancer	2021 (NMPA)
Loncastuximab tesirine (Zynlonta^®^)	CD19	SG3199	Diffuse large B-cell lymphoma High-grade B-cell lymphoma	2021 (FDA) 2022 (EMA)
Tisotumab vedotin (Tivdak^®^)	Tissue Factor	MMAE	Cervical cancer	2021 (FDA) 2025 (EMA)
Mirvetuximab soravtansine (Elahere^®^)	FRα	DM4	Epithelial ovarian, fallopian tube, or primary peritoneal cancer	2022 (FDA) 2024 (EMA)
Sacituzumab tirumotecan (Jiataile^®^)	Trop-2	KL610023	HER2− breast cancer	2024 (NMPA)
Telisotuzumab vedotin (Emrelis^®^)	c-Met	MMAE	Non-squamous non-small cell lung cancer	2025 (FDA)
Datopotamab deruxtecan (Datroway^®^)	Trop-2	DXd	Metastatic, HR+ HER2− breast cancer Non-small cell lung cancer	2025 (FDA/EMA)
Trastuzumab rezetecan (SHR-A1811)	HER2	SHR169265	Non-small cell lung cancer	2025 (NMPA)
Trastuzumab botidotin (A166)	HER2	Duostatin 5	HER2+ breast cancer	2025 (NMPA)
Becotatug vedotin (MRG003)	EGFR	MMAE	Nasopharyngeal carcinoma	2025 (NMPA)
Pivekimab sunirine (Decnupaz^®^)	CD123	DGN462	Blastic plasmacytoid dendritic cell neoplasm	2026 (FDA)

EMA = European Medicines Agency, FDA = Food and Drug Administration, MMAE = Monomethyl auristatin E, MMAF = Monomethyl auristatin F, NMPA = National Medical Products Administration, PE38 = Pseudomonas exotoxin A fragment, * = Withdrawn by both FDA and EMA in 2023.

**Table 3:** Quality assessment of the included trials in respect to predicting therapeutic response to ADC treatment using targeted PET, evaluated according to the QUIPS (Quality In Prognosis Studies) tool.

Potential Bias Domains	Mileva et al. (2024) [21]	Mortimer et al. (2022) [22]	Alhuseinalkhudhur et al. (2023) [23]	Lee et al. (2017) [24]	Lamberts et al. (2016) [26]	Carrasquillo et al. (2019) [27]	Miederer et al. 2026 [25]
**Study participation**							
Source of target population is adequately described	Yes	Yes	Yes	Yes	Yes	Yes	Yes
Methods used to identify population and methods to limit potential bias in recruitment are described	No	No	No	No	No	No	No
Recruitment period is described	Yes	No	Yes	Yes	Yes	No	No
Places of recruitment are described	Yes	No	No	No	Yes	No	No
Inclusion and exclusion criteria are described	Yes	Yes	Yes	Yes	Yes	Yes	Yes
Adequate study participation	Partial	No	No	No	No	No	No
Baseline characteristics of participants are described	Yes	Yes	Yes	Yes	Yes	Yes	Yes
Summary (High/Moderate/Low)	Low	High	Moderate	Moderate	Moderate	High	High
**Study Attrition**							
Proportion of baseline sample available for analysis	Yes	Yes	Yes	Yes	Yes	Yes	Yes
Attempts to collect information on participants who dropped out	No	NA	Yes	NA	NA	No	NA
Reasons and impact of loss to follow-up are provided	Partial	NA	Yes	NA	NA	No	Yes
Description and/or negation of important differences between participants who completed the study and those who did not?	No	NA	Yes	NA	NA	No	No
Summary (High/Moderate/Low)	Moderate	Low	Low	Low	Low	Moderate	Moderate
**Prognostic Factor Measurement**							
Clear definition of prognostic factor	Yes	Yes	Partial	Yes	Yes	Yes	Yes
Valid and reliable measurement of prognostic factor	Yes	Yes	Yes	Yes	Yes	Yes	Yes
Method and setting of prognostic factor measurement	Yes	No	No	No	No	No	Yes
Proportion of data on prognostic factors available for analysis	Yes	Yes	Partial	Partial	Yes	Partial	Yes
Method used for missing data	NA	NA	NA	NA	NA	NA	NA
Summary (High/Moderate/Low)	Low	Moderate	High	High	High	High	Low
**Outcome Measurement**							
Definition of the outcome	Yes	Yes	Yes	Yes	Yes	Yes	Yes
Valid and reliable measurement of outcome	Yes	Yes	Yes	Yes	Yes	Partial	Yes
Method and setting of outcome measurement is the same for all participants	Yes	Yes	Yes	Yes	Yes	Yes	Yes
Summary (High/Moderate/Low)	Low	Low	Low	Low	Low	Moderate	Low
**Study Confounding**							
Important confounders measured	Yes	Partial	Partial	Partial	Partial	Yes	No
Clear definition of confounding factor(s)	No	No	No	No	No	No	No
Valid and reliable measurement of confounders	Unsure	Unsure	Yes	Unsure	Unsure	Unsure	No
The method and setting of confounding measurements are the same for all participants	Unsure	Unsure	Unsure	Unsure	Unsure	Unsure	Partial
Method used for missing data	Unsure	Unsure	Unsure	Unsure	Unsure	Unsure	Unsure
Important potential confounders are accounted for in study design and/or analysis	No	No	Yes	No	No	No	No
Summary (High/Moderate/Low)	High	High	Moderate	High	High	High	Moderate
**Statistical Analysis and Reporting**							
Sufficient presentation of data to assess the analytical strategy	Yes	Yes	Yes	NA	Yes	Yes	Yes
Statistical model and strategy for model building	Yes	Yes	Yes	NA	Yes	Yes	NA
Reporting of results	Yes	Yes	Yes	Yes	Yes	Yes	Yes
Summary (High/Moderate/Low)	Low	Low	Low	Low	Low	Low	Low

The tool presented is slightly simplified in the interest of preserving space. The full QUIPS tool can be accessed via the Cochrane website. ‘Unsure’ is used when no sign of obvious issues is identified, yet the methods are not described in enough detail, such as for measurement of confounders. Colors green, yellow and red indicate low, moderate and high risk of bias, respectively.

## Data Availability

The raw data supporting the conclusions of this article will be made available by the authors on request.

## Supplementary Materials

The following supporting information can be downloaded at https://www.mdpi.com/article/10.3390/cancers18152514/s1, Table S1: PRISMA 2020 Checklist; Table S2: Search strategies for the EMBASE and Web of Science databases; Table S3: Complete PICO statement.
