# Supplementary material for: Predicting Therapeutic Response to Antibody-Drug Conjugates Using Targeted PET Imaging: A Systematic Review
Source: Cancers (Basel). 2026 Aug 5;18(15):2514. doi: 10.3390/cancers18152514 (PMC13464691; doi:10.3390/cancers18152514)
Supplement: Supplementary file 1 [file cancers-18-02514-s001.zip › Supplementary material Tables S2 and S3.pdf]

Predicting Therapeutic Response to Antibody-Drug Conjugates Using Targeted PET Imaging: A Systematic Review

Supplementary Materials

Supplementary Table S2. Search strategies for the EMBASE and Web of Science databases.

EMBASE	'antibody drug conjugate'/exp OR 'antibody drug conjugate' OR 'antibody conjugate'/exp OR 'antibody conjugate' OR 'gemtuzumab' OR 'gemtuzumab ozogamicin' OR 'brentuximab vedotin' OR 'trastuzumab emtansine' OR 'inotuzumab ozogamicin' OR 'moxetumomab pasudotox' OR 'polatuzumab vedotin' OR 'enfortumab vedotin' OR 'trastuzumab deruxtecan' OR 'sacituzumab govitecan' OR 'belantamab mafodotin' OR 'loncastuximab tesirine' OR 'tisotumab vedotin' OR 'mirvetuximab soravtansine' OR 'disitamab vedotin' OR 'cetuximab sarotalocan' OR T-DM1 OR T-Dxd<br>**AND**<br>'positron emission tomography'/exp OR 'positron emission tomography' OR 'positron emission tomography-computed tomography'<br>**AND**<br>'neoplasm'/exp OR 'neoplasm' OR 'malignant neoplasm'/exp OR 'malignant neoplasm'
Web of Science	TS=(ADC OR 'antibody drug conjugate' OR 'antibody conjugate' OR 'gemtuzumab' OR 'gemtuzumab ozogamicin' OR 'brentuximab vedotin' OR 'trastuzumab emtansine' OR 'inotuzumab ozogamicin' OR 'moxetumomab pasudotox' OR 'polatuzumab vedotin' OR 'enfortumab vedotin' OR 'trastuzumab deruxtecan' OR 'sacituzumab govitecan' OR 'belantamab mafodotin' OR 'loncastuximab tesirine' OR 'tisotumab vedotin' OR 'mirvetuximab soravtansine' OR 'disitamab vedotin' OR 'cetuximab sarotalocan')<br>**AND**<br>TS=(Neoplasms OR Cancer)<br>**AND**<br>TS=('Positron-Emission Tomography' OR 'Positron Emission Tomography Computed Tomography' OR PET) and English (Languages) and Humans (MeSH Headings)

Supplementary Table S3. Complete PICO statement.

PICO	Inclusion Criteria	Exclusion Criteria
Population	Patients undergoing cancer treatment with ADC	
Intervention	PET preformed utilizing a tracer targeting the same antigen.	Only PDG-PET performed.
Comparison	No relevant Comparison element was identified.	
Outcomes	Sensitivity, specificity, PPV, and NPV in relation to lesion response determined by either RECIST 1.0/1.1, variations in SUV measurements (total, maximum, peak, mean), or outcomes like OS, PFS, and TTF.<br><br>In case of neoadjuvant treatment; any measurement of tumor response in the pathology report or outcomes like OS or PFS.	No follow-up.<br><br>Outcomes related to physician choice.
